# Human emotional evaluation of ancestral and modern threats: fear, disgust, and anger

**DOI:** 10.3389/fpsyg.2023.1321053

**Published:** 2024-01-04

**Authors:** Šárka Peléšková, Jakub Polák, Markéta Janovcová, Aleksandra Chomik, Kristýna Sedláčková, Daniel Frynta, Eva Landová

**Affiliations:** ^1^Department of Zoology, Faculty of Science, Charles University, Prague, Czechia; ^2^Department of Economy and Management, Ambis University, Prague, Czechia; ^3^National Institute of Mental Health, Klecany, Czechia

**Keywords:** anger, COVID-19, fear of heights, fear of snakes, ontogenetic threat, oral disgust, pandemic of airborne disease, phylogenetic threat

## Abstract

**Introduction:**

Animal and human ancestors developed complex physiological and behavioral response systems to cope with two types of threats: immediate physical harm from predators or conspecifics, triggering fear, and the risk of infections from parasites and pathogens leading to the evolution of the behavioral immune system with disgust as the key emotion. Integration of the evolutionary concepts of the fear module and behavioral immune systems has been infrequent, despite the significant survival advantages of disgust in various contexts. Studies comparing attention to ancestral and modern threats accompanied by fear have yielded ambiguous results and what qualifies as salient modern disgusting stimuli remains unclear. We do not know whether disgust or the behavioral immune system, as inherent aspects of human psychology, have adapted to safeguard us from pandemic risks or poisoning by modern toxic substances.

**Methods:**

To test these effects, we have developed a survey comprised of 60 short vignettes describing threats evoking fear and disgust belonging to one of the three main categories of threats: (1) ancestral (phylogenetic), (2) modern (ontogenetic), and (3) pandemics of airborne disease. Each vignette was evaluated on a 7-point Likert scale based on fear, disgust, and anger. In total, 660 respondents completed the survey. The data were analysed using a factor analysis and general linear model with the respondent as a random factor.

**Results:**

The results show that the strongest fear is triggered by modern threats (electricity, car accidents), while the highest disgust is evoked by ancient threats (body waste products, worms, etc.). Interestingly, disgust does not respond to modern threat stimuli such as toxic substances or radioactivity as these evoke mainly fear and anger. Finally, a distinct response pattern was found for pandemic threats, in which both fear (e.g., of disease and death) and disgust (e.g., of used face masks) are employed.

**Discussion:**

Our study offers valuable insights into the emotional responses to ancestral and modern threats and their adaptation to pandemic challenges. Ancestral threats are not always more powerful stimuli than adequate threats of the modern type, but they function specifically. Thus, snakes and heights as fear-inducing ancestral threats form separate factors in a multivariate analysis, whereas all ancestral disgust stimuli group together. The threat of a pandemic forms a specific category and people process it emotionally and cognitively. These insights contribute to our understanding of human psychology and behavior in an ever-changing world.

## 1 Introduction

Throughout human evolution, the survival of our animal and human ancestors was perpetually challenged by diverse environmental threats (Öhman, 2007). These encompassed immediate physical dangers from predators and conspecifics belonging to other tribes (Barrett, 2015), as well as insidious risks posed by parasites and pathogens (Perry, 2014). Research in evolutionary psychology suggests that such threats that were likely to cause injury or even death have shaped the human brain’s fear response, resulting in the development of cognitive mechanisms that prioritized survival (Öhman and Mineka, 2001).

The amygdala as a key component of the brain’s fear circuitry played a primordial role in the detection of phylogenetic threats (LeDoux, 2003; Öhman, 2005) and the initiation of a rapid and instinctual “fight-or-flight” response (LeDoux, 2012). Furthermore, the ancestral environment fostered the development of fear-learning mechanisms (Öhman and Mineka, 2001; Mineka and Zinbarg, 2006; Zsido et al., 2023), enhancing the acquisition of threat-related information for adaptive decision-making. Interestingly, the distribution of fears is non-random as some objects or situations tend to be feared by humans much more often than others (especially animals such as snakes or spiders and natural/physical elements such as heights, storms, dark, enclosed spaces etc.; Curtis et al., 1998). Therefore, Seligman (1971) proposed an influential theoretical model of biological preparedness arguing that phobic reactions reflect our evolutionary past and are associated with stimuli posing a real threat to the survival of human pre-technological ancestors (see also Bracha, 2006).

However, others have challenged the view of the amygdala being a fear module responding specifically to fear-related stimuli and argued that research has already shown a variety of triggers of the amygdala activation including positive stimuli. Therefore, Sander et al. (2003) proposed an alternative theory that the amygdala processes objects or situations that might be relevant to the organism no matter its emotional valence.

Apart from the fear-inducing predators and conspecifics, another critical danger has existed throughout our evolutionary history, representing an even more substantial threat - the risk of infection from parasites, bacteria, and viruses (Curtis, 2014). However, given the qualitative distinction between imminent physical attacks and pathogen exposure, the emotion of fear might not have been the only appropriate response (Oaten et al., 2009). Instead, our ancestors, even as early mammals, evolved a specialized mechanism known as the behavioral immune system, with disgust as its key emotion (Curtis and Biran, 2001; Schaller and Park, 2011). Whether disgust evolved from a simple response to bad taste (distaste), which can indicate spoiled and potentially dangerous food (Chapman et al., 2009; Rozin et al., 2009), or whether it was designed from the beginning to respond to a wider range of stimuli associated with disease and infection (Curtis, 2014), the authors agree that the category of triggers has been further expanded throughout biological and cultural evolution, including even immoral acts (moral disgust; Tybur et al., 2013).

Disgust in any case serves as a powerful signal to avoid potential sources of infection, supporting the survival of our ancestors in pathogen-rich environments. Behavioral responses to disgust include withdrawal, distancing, or dropping of the potentially infectious object (Curtis et al., 2011). Universal disgust elicitors are bodily wastes and fluids (faeces, urine, vomit, blood, saliva, mucous), organs, sick or unhygienic individuals, spoiled or unfamiliar food, and certain animals acting as disease vectors (Tybur et al., 2013).

Functionally, both fear and disgust serve to protect the biological integrity of an organism (Nesse, 1990) but are principally different as to the characteristics of impending danger (Keltner and Gross, 1999). Disgust, in contrast to fear, activates at different levels a neural network involving the anterior insular cortex, basal ganglia, ventrolateral and medial prefrontal cortex, anterior temporal cortex, and visual cortex (Wicker et al., 2003; Chapman and Anderson, 2012; Koenigs, 2013; Becker et al., 2016). As for the physiological response, disgust is usually associated with activation of the parasympathetic nervous system, including heart rate deceleration (Cisler et al., 2009), however, the results of physiological studies are not always consistent. Kreibig (2010) in her review suggests a second, partially overlapping, pattern characterized by sympathetic-parasympathetic co-activation with heart rate acceleration, faster breathing, and decreased inspiration (in relation to contamination stimuli, in contrast to blood and injury). In conclusion, there is an ongoing debate about parasympathetic activation in disgust reaction, but it is clearly not as strong a sympathetic activator as fear (Rozin et al., 2016).

While much research has focused on disgust in humans, studies in non-human primates have also provided valuable insights into the evolutionary origins and function of disgust and the behavioral immune system (Rottman, 2014). In primates, the facial expression of disgust is characterized by distinct features, such as a raised upper lip, exposing teeth, a wrinkled nose, and narrowed eyes (Preuschoft and van Hooff, 1995). These facial movements serve as important communicative signals within primate social groups. It has been shown that a group of mandrills exhibits a reduced tendency to remain in close proximity (<1 m) to a highly parasitized faecal sample (Poirotte et al., 2017). Given that the divergence time between Cercopithecoidea (Old World monkeys, a superfamily containing mandrills) and Hominoidea was estimated to be the Oligocene period (33.9–23 MYA; Springer et al., 2012), the disgust must have emerged even earlier in primate evolution.

In a series of experiments with bonobos, researchers observed that these primates exhibited avoidance behaviors and contamination-risk sensitivity in response to food items along a gradient of contamination probabilities. These responses appeared to require multisensory cues to associate contamination events with specific food items, aligning with the parasite avoidance theory of disgust. Surprisingly, there was no observed sex-based bias in contamination-risk aversion, and the study suggests that physiological responses to contaminants may have evolved alongside behavioral avoidance mechanisms in primates (Sarabian et al., 2018). Similarly, the feeding behavior of chimpanzees (*Pan troglodytes troglodytes*) is influenced by potential contaminants, primarily conspecific faeces. When food was associated with the odour of faeces, these animals were less inclined to feed and often vacated the area. Conversely, there was no discernible difference in their feeding behavior when exposed to the odours of blood or semen, which are not necessarily linked to pathogen avoidance but could be related to antipredator behavior or reactions to conspecific aggression (Sarabian et al., 2017; see a review by Schwambergová et al., 2023).

Over time, the nature of threats has evolved, ranging from ancestral challenges that early humans faced to the modern complexities of the contemporary world. Only a few studies compared the evaluation of ancestral and modern threats. Shapouri et al. (2023) have recently demonstrated that the evolutionary age of disasters is one of the factors that affect emotional experiences evoked by these threats and can impact our evaluations of catastrophes. Technological (modern, manmade) disasters were rated as slightly less arousing but significantly more unpleasant than natural (ancient) disasters. In another study, people were more concerned about the negative consequences of human hazards compared with natural hazards. The same negative outcome (e.g., number of birds killed by an oil spill) was more negatively evaluated when caused by humans than when caused by nature. Furthermore, when identical risk information was provided, participants evaluated nuclear power more negatively compared with solar power (Siegrist and Sütterlin, 2014).

While ancestral life-threatening stimuli are strong attention-catchers (Öhman et al., 2001; Blanchette, 2006; Rudolfová et al., 2022; Štolhoferová et al., 2023), the impact of modern threats on attention remains equivocal (*cf.* Zsido et al., 2019; Abado et al., 2023). Despite the differences in cognitive processing, both ancestral and modern threats involve the activation of stress-responsive systems. The hypothalamic–pituitary–adrenal (HPA) axis releases cortisol, facilitating adaptive physiological responses to threats (McEwen and Gianaros, 2011). Additionally, the role of the amygdala in detecting threat-related stimuli remains relevant across both contexts, emphasizing its evolutionary significance (Phelps and LeDoux, 2005). However, the distinct cognitive evaluation and processing of modern threats may modulate the extent to which these shared neural and physiological pathways are engaged (Öhman and Mineka, 2001).

The coexistence of ancestral and modern threats in the contemporary world has also implications for mental health and well-being (Katsampouris et al., 2022). An overactive fear response such as specific phobias, initially adaptive for ancestral threats, may contribute to anxiety disorders when chronically activated in response to modern stressors (Nesse, 1999). The adaptation of ancestral fear mechanisms to modern threats may exacerbate the experience of chronic stress and anxiety (McEwen and Gianaros, 2011). Furthermore, the ubiquity of modern threats in media may amplify fear responses and contribute to heightened levels of anxiety and stress-related disorders (Vasterman et al., 2005).

To the best of our knowledge, no similar research comparing a response pattern to ancestral and modern disgust elicitors exists. Moreover, it is not even known whether there are any contemporary threats (except moral code violations) with the potential to trigger disgust. Only recently, a study by Hacquin et al. (2022) showed that nuclear energy might be a modern disgust elicitor activating the behavioral immune system. One of the main goals of our study was to support that finding and test, whether other modern disgusting stimuli could be identified. We aimed to create “mirror” stimuli similar to fear studies, which compare, for example, fear of snakes and fear of guns (for a review, see for example Shapouri and Martin, 2022), i.e., fear of injury or death, which might however be caused by stimuli of different evolutionary age. Thus, in the case of disgust, we aimed to create situations in which poisoning might occur using stimuli such as spoiled food (ancestral) versus toxic chemical substances (modern; see below).

Another possible candidate is the threat of a pandemic of infectious disease. In the past and even in modern times, infectious diseases have remained a significant cause of mortality, especially in lower-middle-income countries. Pandemics have been a recurring phenomenon throughout human history, shaping societies, economies, and healthcare systems and adherence to avoidance behavior and good hygiene practices, driven by disgust, can mitigate the risk of infection (Tybur et al., 2013).

The types of diseases more prevalent in human evolutionary history, which shaped our disgust response, might differ from those that pose a threat today. Since for most of their evolutionary history humans lived in relatively small groups and with only limited inter-group contacts (Weisdorf, 2005), one should expect only epidemics with local character. Mainly the transmission of airborne diseases via respiratory droplets and aerosols depends heavily on human mobility and contact frequency within and between populations (Kucharski et al., 2020). Human ancestors, living in relatively small groups with limited inter-group contacts, were more susceptible to local epidemics rather than global pandemics caused by airborne diseases (Weisdorf, 2005; Troisi, 2020). However, approximately 10,000 years ago, with the formation of cities and extensive trade networks, the landscape of disease transmission changed (Weisdorf, 2005). During Antiquity, we know of several large pandemics, e.g., the Antonine plague (suspected smallpox pandemic; Duncan-Jones, 1996) or the Plague of Justinian (bubonic plague pandemic; Frith, 2012). Since the Middle Ages well into the 19th century, repeated outbreaks of bubonic plague and smallpox were the cause of hundreds of millions of deaths worldwide (Frith, 2012). One of the most devastating pandemics in history, the Black Death, caused by the bacterium *Yersinia pestis*, decimated Europe’s population between 1,347 and 1,351. The rapid spread of the disease through fleas on rats led to millions of deaths, profoundly affecting medieval societies (Cohn Jr, 2002). The second largest pandemic yet is considered the so-called Spanish flu of 1918–1920 (Trilla et al., 2008). Caused by the H1N1 influenza virus, the Spanish Flu is often cited as a benchmark for pandemic severity. With an estimated 50 million deaths worldwide, its impact was magnified by the context of World War I and the global movement of troops (Barry, 2009). Interestingly, all these historically deadly pandemics were of airborne diseases.

However, it remains a topic of inquiry as to how disgust may reduce the risk of infections transmitted through the respiratory route, such as tuberculosis or viral influenzas (Schwambergová et al., 2023). Yet, sudden outbreaks of bacterial or viral diseases with the potential to rapidly spread globally present one of the biggest health challenges humans will need to face in the future. This is especially the case of airborne pathogens as we witnessed recently with the pandemic of COVID-19 (Wu et al., 2020). In conclusion, the threat of a pandemic can be considered as a modern threat against which both emotions of fear and disgust could protect. This has also become one of our main research interests here.

Fear and disgust are not the only emotions triggered by certain types of threats. The COVID-19 pandemic, as mentioned above, has brought unprecedented challenges and disruptions to societies worldwide (Cash and Patel, 2020). While much attention has been focused on the physical health consequences of the virus, there is growing recognition of the impact of the pandemic on mental health (Cullen et al., 2020). The COVID-19 pandemic has engendered a profound sense of uncertainty and fear due to its rapid spread, high mortality rates, and the lack of a definitive treatment or vaccine during the initial phases (Taylor, 2019; Coelho et al., 2020). This has been further accentuated by multiple psychosocial stressors associated with the pandemic such as economic instability, job loss, and financial strains, creating fertile ground for increased anger to manifest (Pfefferbaum and North, 2020). Finally, social isolation, disrupted routines, and concerns about loved ones’ health have added to the emotional burden (Brooks et al., 2020). These stressors can amplify frustration and irritation, leading to anger as an emotional outlet. Thus, anger has emerged as a significant and complex emotion during this crisis (Smith et al., 2021) and needs to be incorporated into psychobehavioral studies of pandemic threats.

However, anger is not only an emotional response to various stressors like restraint from many “normal” goal-directed activities caused by the pandemic situation, but it is one of the basic emotions that informs and guides many aspects of human behavior (Scarantino and Griffiths, 2011). There is neuroscientific evidence that points to the phylogenetic origins of two circuits underlying anger that have had an evolutionary role in promoting the survival of human ancestors (reviewed in Williams, 2017). This emotion is tightly connected with approach-avoidance motivation and serves as an internal signal helping to overcome different types of obstacles and aversive situations. External displays of anger can be cross-culturally stable (Matsumoto et al., 2010) and are a communication signal that plays an important role in dealing with conflicts in interpersonal relationships and emotional attachments (Williams, 2017). Anger motivates humans mostly to approach the threat and deal with it, whereas fear and disgust are linked more with an avoidance response (Harmon-Jones et al., 2013). From an evolutionary point of view, we can see the ancestral function of anger for confronting various threats, and thus this emotion can easily supplement an evasive function of fear whenever human ancestors had to face the imminent danger of predation or attacks from conspecifics.

There is research on the theory of biological preparedness when scientists use angry faces as ancestral stimuli (similarly to snakes or spiders used in research on the evolution of fear) to trigger anger and show how it works in the context of conditioning. It is predicted that evolutionarily prepared stimuli should be conditioned faster, and their extinction should be slower. Moreover, the psychophysiological response to them should be stronger compared to neutral stimuli (Öhman and Dimberg, 1978; McNally, 1987; Ney et al., 2022).

Interestingly, humans generalize anger also to moral indignation over a violation of morality that is caused by the wrongness of one’s actions and especially by the intent to harm (Hechler and Kessler, 2018). Surprisingly, the same moral violation of the rules is experienced by some people more as anger, while other people report feeling disgusted. Feeling disgusted at moral violations is more likely to occur whenever others break the rules and is more likely to be associated with indirect aggression. Feelings of anger are typical when the respondent himself violates the moral code (Molho et al., 2017). Moreover, moral anger and moral disgust appear to have a surprisingly similar pattern of activation in fMRI (Oaten et al., 2018). Anger for its evolutionary importance as well as for its generalization to dealing with moral violations is an important emotion that should accompany or complement our emotional reaction to ancestral as well as modern threats.

### 1.1 Aims

While fear and disgust have been extensively studied separately, there is a need to explore them simultaneously. It is necessary as well to compare ancestral and modern threats to understand the intensity of emotional and behavioral responses they trigger and their adaptability in the context of modern challenges like pandemics. Integration of the evolutionary concepts of the fear module and behavioral immune system has been infrequent, despite the significant survival advantages of disgust in various contexts. Studies comparing attention to ancestral and modern threats accompanied by fear yielded ambiguous results and what qualifies as salient modern disgusting stimuli remains unclear. We do not know whether disgust or the behavioral immune system, as inherent aspects of human psychology, have adapted to protect us from pandemic risks or poisoning by modern toxic substances. This paper explores the foundations of fear and disgust in the context of both ancestral and modern threats, elucidating their emotional manifestations and potential relevance to modern challenges.

The specific aims of the study were to find out whether:

1. There is a difference between ancestral and modern threats within each emotion, in other words, if the ancestral machinery can be effectively applied to new types of current threats or which threats, either phylogenetic or ontogenetic, are more salient in triggering fear, disgust, or anger.
2. There are specific triggers (types of threats) of each emotion or it is rather the current level of threat relevance that is primordial. Or, what stimuli are the best triggers of fear, disgust, or anger?
3. The psychobehavioral circuits for processing fear and disgust have been adapted to respond to threats of pandemics of various diseases and whether these elicit more fear, disgust, or anger.

## 2 Materials and methods

### 2.1 Respondents

In total, 660 respondents completed the whole survey (484 women, 176 men). The participants were of Central European origin and spoke Czech. We recruited them mainly from the staff and students at several universities (including a University of the Third Age) and their relatives, so that we could obtain respondents of different age groups but with the same socioeconomic background (age 18–88, mean 39.98 ± 18.47). Out of these, 295 participants have had a biological education (*sensu lato*, including medicine, or agriculture), while the remaining 365 participants have been educated in a different field (mainly technical or social sciences).

Biological education is a process usually involving dealing with various animals (vertebrate and invertebrate) and using various methods from microscopy to handling living organisms or dissecting the dead ones. For biological students, all these activities are initially more or less disgusting like for other people. However, the disgust sensitivity is lower for university students with more interest and higher competencies (Randler et al., 2013). However, increased interest and decreased disgust sensitivity are also measurable for similar activities with 10 to 12-year-old children (Prokop and Fančovičová, 2017). Eventually, all students become accustomed to various animal-related practices during the educational process, not only with respect to reducing disgust but also fear of unpopular animals, both of which are significantly reduced (Randler et al., 2012). Among biologically educated respondents (biologists, biology teachers, physicians, nurses, and people with agricultural education at high school or college degree), we repeatedly found a lower disgust propensity and lower fear of fear-inducing animals such as snakes (fear: Rádlová et al., 2020; fear and disgust: Polák et al., 2020a, 2022; Staňková et al., 2021).

In our previous studies, we have often detected the effect of gender on fear and disgust (women experiencing higher fear of snakes: Polák et al., 2016 and spiders: Polák et al., 2020b; or higher disgust propensity: Polák et al., 2019). The effect of age (decreasing emotional sensitivity with age) on the subjective experience of fear and disgust when evaluating animal stimuli or completing assessments is less pronounced than the effect of gender but should still be considered (Landová et al., 2018; Polák et al., 2020a, 2022). As a significant proportion of vignettes simulating ancestral threats focus on snakes or invertebrates, we find it necessary to include the effect of biological education, sex, and age in the statistical models.

### 2.2 Stimuli and procedure

During a pilot study, we developed 77 short vignettes describing potentially dangerous situations that might evoke strong fear or disgust. We did not include vignettes on anger for several reasons. First, it is not clear what stimuli should be ancestral and modern concerning anger. Second, we see anger rather as a complementary emotion to fear and tightly attached to the moral aspect of disgust. Finally, one of the main objectives of this study was to see if the pandemics of airborne disease would be more similar to ancestral or modern threats based on fear and disgust – both emotions are linked to avoidance behavior, which may be also useful during the pandemic threat. As anger often leads to the opposite behavior, i.e., approach and attack, it would be complicated to think about its evolutionary advantage in the context of pandemic threats.

When creating the vignettes, we took inspiration from several established questionnaires, e.g., the Snake Questionnaire (Klorman et al., 1974, Czech translation by Polák et al., 2016) and the Disgust Scale - Revised (Haidt et al., 1994, modified by Olatunji et al., 2007, Czech translation by Polák et al., 2019), however, we modified the questions to be more relevant for Czech respondents, local environment, and their everyday lives. Most of the vignettes were newly created.

Each vignette belonged to one of the three main categories of threats: (1) ancestral (phylogenetic; snakes, heights, spoiled food, or other contamination disgust, e.g., “I go to the basement to get something and suddenly I hear a snake hissing.”), (2) modern (ontogenetic; electricity, car accidents, toxic chemical substances visible and invisible, e.g., “I’m riding as a passenger in a car when suddenly the driver loses consciousness.”), and (3) pandemics of an airborne pathogen (COVID-19 or another unspecified disease, e.g., “I feel someone sneeze on my face.”). In total, 112 participants rated each vignette on a 7-point Likert scale by fear, disgust and anger during the pilot study. Based on these ratings, we selected only those vignettes that strongly elicited exclusively one of the two main negative emotions (high fear and low disgust and vice versa; here, we consider anger to be rather a secondary emotion). 17 vignettes evoking weak emotions or vignettes with ratings that did not correspond well to the predefined category were excluded from the main study.

Thus, 60 vignettes have been retained for further testing, 20 for each threat category (for the stimuli examples, see Table 1, and for the full list of vignettes, including the excluded ones, see Supplementary Table S1). The data collection took place between October 2022 and June 2023.

**Table 1:** Examples of the vignettes used in the experiment.

Vignette category	Expected predominant emotion	Vignette example
Ancestral	Fear	I’m camping in nature and see a snake slithering near my tent.
	Disgust	I urgently need to use the toilet on the train, but it is very dirty.
Modern	Fear	I’m driving a car in the winter, and I feel that I am losing control of the vehicle on an icy road.
	Disgust	While swimming in a river, I find that there is an iridescent oil coating on the surface that has an unpleasant chemical smell.
Pandemic	Fear	A close family member is in the ICU with a severe case of respiratory disease.
	Disgust	A person with obvious symptoms of respiratory disease sits down next to me on public transport.

As the extent of the study did not allow to cover all possible situations that people may be afraid of, we included two open-ended questions at the very end of the experiment, where we asked what they currently feared the most or what they found the most disgusting. The participants were instructed to write their answers down if these stimuli were not represented in the questionnaire (no maximum stimuli limit was given and the respondents could also leave the question unanswered).

The testing procedure was conducted both online and as pen-and-paper. While younger participants usually prefer the online format, older people are easier to recruit in person, we were thus able to obtain a more age-balanced sample. The respondents were first asked a series of sociodemographic questions. Each vignette was then evaluated on a 7-point Likert scale based on fear, disgust, and anger (1 = not at all, 7 = extremely strong). The participants were asked to rate all the vignettes according to all three emotions, no time limit was set for the task.

### 2.3 Ethical note

This study was carried out following the approval of the Ethical Committees of Charles University, Faculty of Science (approval no. 2021/02, granted on 14 April 2021) and National Institute of Mental Health (no. 91/21, granted 31 March 2021) and in accordance with the Declaration of Helsinki. All subjects provided their informed consent with participation in the study and personal data processing.

### 2.4 Statistical analysis

Raw scores for each question were used where possible, as we attempted not to transform the data to maintain as much variability as possible in the ratings of individual respondents. Agreement in the emotional evaluation among the respondents was quantified using the Kendall’s coefficient of concordance (performed in SPSS 22; IBM Corp, 2013). Cumulative link mixed models for ordinal data (CLMM as implemented in R package ordinal; Christensen, 2022) were computed to examine the effect of respondents’ characteristics (gender, age, education) and stimuli categories on the evaluation of vignettes on a Likert-like scale; respondents’ identity was introduced as a random factor. To test the significance of differences in emotional evaluation between different stimuli categories, we performed a *post hoc* Tukey test (using the R package lsmeans; Lenth, 2016). Subsequently, a factor structure in the vignette ratings was examined using a factor analysis (principal component extraction and varimax normalized rotation method were used). A parallel analysis was used to determine the number of factors. We then visualized the data structure using a cluster analysis (the distance matrix was calculated using Pearson correlations among ratings, and tree diagrams were built using the Ward’s method). We also applied the item response theory (IRT) approach to the vignettes’ ratings, specifically a graded response model to check for the discrimination parameter. This was performed in Stata 18 (Stata Corp, 2023). Unless otherwise stated, the calculations were performed in R Statistical Software (v. 3.6.1; R Core Team, 2019) and Statistica 10 (Stat Soft, Inc., 2011).

## 3 Results

### 3.1 Emotional salience of stimuli

Six hundred and sixty respondents rated 60 vignettes describing a potential threat on a 7-point scale according to three negative emotions: fear, disgust, and anger. One hundred and twelve of these respondents participated in the pilot study evaluation, where they evaluated a larger number (77) of vignettes. However, because the ratings of the final set of vignettes obtained from these two experiments are highly correlated (Spearman’s correlations for fear R = 0.962, disgust R = 0.923 and anger R = 0.965, all *p* < 0.0001), we pooled the two samples of respondents for all subsequent analyses.

**Figure 1:**
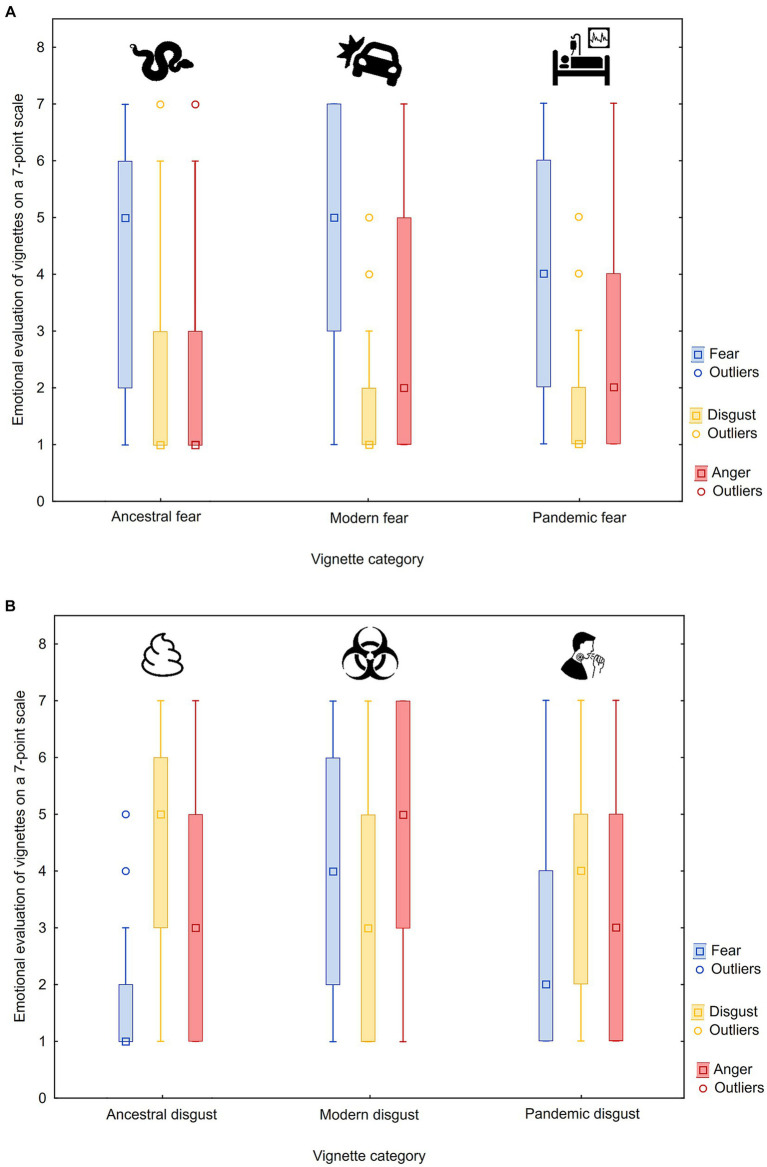
Graphical representation of fear, disgust, and anger ratings of vignettes representing potential threat for humans (using raw scores). Six vignette categories are divided into two graphs for a better clarity: fear vignettes **(A)** and disgust vignettes **(B)** according to the expected predominant negative emotion. Median (middle point), lower and upper quartiles (box range) and non-outlier minimum and maximum values (whiskers) are provided together with outlier points.

Mean fear and disgust scores were negatively correlated (Kendall’s τ = −0.497, *p* < 0.0001), while the mean disgust and anger scores were correlated positively (Kendall’s τ = 0.311, *p* = 0.0004). Correlations between the fear and anger scores were not significant but there was a trend for a negative relationship suggesting the dichotomy between a fight or flight response. For mean emotional ratings in each vignette category, see Table 2, and for a graphical representation, see Figure 1.

**Table 2:** Mean ratings of fear, disgust, and anger for individual categories of threats as described by short vignettes (7-point scale, 1 = not at all, 7 = extremely strong).

Category	Mean fear	Fear SD	Mean disgust	Disgust SD	Mean anger	Anger SD
Ancestral fear	**4.330**	2.13	2.103	1.78	2.119	1.80
Modern fear	**4.820**	2.02	1.886	1.62	3.095	2.14
Pandemic fear	**3.930**	2.08	1.916	1.59	2.659	2.03
Ancestral disgust	2.002	1.67	**4.407**	2.03	3.503	2.17
Modern disgust	3.900	2.16	3.167	2.07	**4.432**	2.15
Pandemic disgust	2.497	1.89	**3.749**	2.06	3.456	2.12

The strongest emotion in each category is indicated in bold, standard deviations (SD) are also provided.

For most stimuli, the predominant emotion (highest score) corresponded to the pre-defined category, i.e., fear vignettes elicited high fear and low disgust and vice versa. The only exception was for the modern disgust category, where fear was stronger than disgust and the highest scores were found in the anger evaluation.

While comparing ancestral versus modern stimuli, our results do not suggest that ancestral threats should universally be more powerful than adequate threats of modern type. While the highest disgust is evoked by ancient threats (body waste products, worms, etc.), the strongest fear is triggered by modern threats (electricity, car accidents).

### 3.2 Agreement among respondents

Despite high variability in stimuli and respondents, the evaluation agreement was significant and quite high: Kendall‘s coefficient of concordance for fear W = 0.408, disgust 0.378, and anger 0.346 (all *p* < 0.0001). Interestingly, there was a higher agreement for ancestral vignettes (ranging from 0.478 to 0.409) for all emotions compared to both modern (0.319 to 0.300) and pandemic threats (0.382 to 0.245) when computed separately (all *p* < 0.0001).

### 3.3 Variability among respondents

Next, we performed generalized linear models (GEEGLM) to analyse the effect of respondents’ characteristics (gender, age, biological education) and stimuli threat categories on the emotional evaluation of vignettes. Raw scores of individual respondents were used as a response variable, and respondents’ identity was introduced as a random factor. The results showed that all explanatory variables had a significant effect on the evaluation of fear (gender and category *p* < 0.0001, age *p* = 0.0078, education *p* = 0.0028) and disgust (gender, education and category *p* < 0.0001, age *p* = 0.0042). For anger evaluation, the effect of gender, education, and category (except one case of pandemic disgust category) was significant (all significant *p* < 0.0001), while the effect of age was not significant. Thus, there was a slight tendency for higher scores in women and respondents with non-biological education for all three emotions and higher scores in older people for fear evaluation and lower scores in older people for disgust evaluation. However, these effects of respondents’ characteristics were rather subtle compared to the effect of stimulus belonging to a category (for complete results, see Supplementary Table S2).

### 3.4 Factor analysis and item response theory

Since the threat category effect was the strongest of all explanatory variables examined for all emotions, we looked at this variable in more detail. At first, we performed a *post hoc* Tukey test for the differences between all six pre-defined stimuli categories (pairwise comparisons). All of the comparisons were significant (on the *p* < 0.0001 level) except for one pair in each emotional evaluation: there was no difference in fear scores between modern disgust and pandemic fear vignettes, no difference in disgust ratings between modern fear and pandemic fear and no difference in anger ratings between ancestral disgust and pandemic disgust.

Subsequently, a factor structure in the vignette ratings was examined using a factor analysis, number of factors was determined by a parallel analysis. As for fear, five separate factors were recognized and together explained 36.58 of the total variability. The first factor consisted of most of the disgust-related vignettes (i.e., low fear group), the second one grouped together most of the fear vignettes except for snake fear (separate factor 3) and the majority of pandemic fears (factor 4). For disgust, six factors explained 35.52% of the total variability and the grouping of vignettes corresponded quite well to the pre-defined categories, except for the ancestral fear category – snakes once again formed a distinct cluster (factor 4), while fear of heights grouped with pandemic fear or modern fear vignettes. Although the modern disgust category elicited some level of fear, it did not group with other fear-related vignettes. And for anger, four factors explained 31.04% of the total variance. Pandemic disgust and most of modern disgust vignettes formed their respective distinct factors (factor 3 and 4), the remaining factors consisted of the rest of the disgust-related (factor 1) and fear-related (factor 2) vignettes (for all factor loadings, see Supplementary Table S3, and for the visualization, see Supplementary Figure S1).

Finally, we applied the item response theory (IRT) approach to the vignettes’ ratings, specifically a graded response model to check for the discrimination parameter. The higher the coefficient, the more the item discriminates between respondents. On the other hand, a low discrimination coefficient might also be interpreted as high agreement between respondents. Thus, both results might be relevant in our experiment. To further examine the results, we then computed Spearman’s correlations between the discrimination coefficient and mean emotional ratings of each item in each vignette category. Here, we briefly describe the most important results (please see Supplementary Table S4 for complete results). For fear, most correlations were negative (higher fear rating, lower discrimination) except for the modern fear category. For disgust, none of the correlations was significant (on the *p* < 0.05 level). For anger, the correlations within fear categories were also negative, and disgust categories were not significant. This pattern might indicate some differences between fear and disgust evaluation which will be further discussed below.

### 3.5 Open-ended questions

As the extent of the study did not allow to cover all possible situations that people might be afraid of, the respondents had the opportunity to express themselves in optional open-ended questions at the very end of the experiment. Due to the nature of this optional questions, the responses have not been statistically processed in depth, but we present some interesting findings. The three most frequently mentioned fears were: fear of war (including specifically the war in Ukraine, Russian aggression, or the threat of nuclear war; 112 respondents), fear for the life and health of family or loved ones (110 respondents), and fear for one’s future (most often fear of not finishing school, exams, not being able to find a job, etc.; 81 respondents). While the first two categories were rather evenly represented across all ages, fear of the future was more prevalent among younger respondents. Considering the age factor and frequency of the answer “war,” these responses could be taken as a reflection of what is currently happening in the respondents’ lives, i.e., they would represent a currently relevant threat.

For disgust, there were generally fewer stimuli not covered by the main questionnaire. Immoral behavior (e.g., lying, recklessness, or selfishness; 77 respondents) was the most frequently mentioned, followed by poor hygiene (e.g., bad human smell; 62 respondents), and spiders (52 respondents) came in third; thus, rather ancestral stimuli appeared. There was no obvious age pattern, but poor hygiene and spiders were strongly prevalent among women (men were more likely to leave the question unanswered). The difference in responses regarding fear and disgust in the open-ended questions reflects the main results from the vignette assessment.

## 4 Discussion

Understanding the complex interplay between ancestral and modern threats and their impact on human emotional responses is crucial for unraveling the intricacies of human psychology. This section focuses on three key points derived from our research, which will be discussed in detail in an order corresponding to the aims of the study: the difference between ancestral and modern threats within each emotion, stimulus specificity and triggering of individual emotions, and the adaptation of psychobehavioral circuits to pandemic threats.

### 4.1 Emotional evaluation of ancestral and modern threats

The complexity of human emotional responses to various threats is a central theme that emerges from this study. While the evolutionary perspective suggests that ancestral threats should elicit more intense emotional reactions due to their historical relevance, or conversely, modern threats could be considered more pertinent in today’s world, this research reveals a nuanced interplay of emotions (Öhman, 2007). Surprisingly, our findings indicated that the emotional salience of threats did not always align with their categorization as ancestral or modern.

The rather unexpected dominance of fear in response to modern threats, such as car crashes and electricity, challenges conventional wisdom (LeDoux, 2003) and the expectation that ancestral threats should universally elicit stronger emotions (Seligman, 1971; Öhman, 2007). It suggests that the immediacy and potential for physical harm associated with these threats can trigger a powerful fear response, overriding other emotional considerations. In today’s fast-paced and technology-driven world, where these modern threats are ever-present, our evolved fear response may be adapting to prioritize immediate physical safety (LeDoux, 2012), i.e., prioritize currently relevant threats over the evolutionary older ones. This fits well into the neuropsychological ‘relevance theory’ first proposed by Sander et al. (2003). These authors hypothesize that although the amygdala might have been originally shaped to respond exclusively to various threats via a fear psychophysiological reaction and defensive behavior, it has then evolved into a less specialized system processing and labelling all stimuli relevant to the goals and needs of an organism.

Nevertheless, snakes and heights, representing ancestral fears (Seligman, 1971; Nesse, 1994), ranked as the second most feared stimuli in our experiment, underscoring the enduring impact of these threats on human psychology. It is worth noting that 1.3 million people die each year because of road traffic crashes compared to 80 to 140 thousand of people dying because of snake bites (according to World Health Organization, 2022; World Health Organization, 2023), while snake phobia is more prevalent (2.6%; Polák et al., 2016) than phobic fear of driving (1.1%; Becker et al., 2007). Phobias of various animal species also do not always correspond to the fear of people in the general population, nor the actual danger of the feared animals (see for example Polák et al., 2020a; Staňková et al., 2021). This further highlights the complexity of human fears and the importance of experimental design when comparing different stimuli.

Pandemic threats (connected with fear of severe course of illness, suffering, and death) ranked third in terms of fear, with still high ratings, thus reflecting their relevance in contemporary times. Although there are studies that have addressed the fear emotion induced by COVID-19 (e.g., Ahorsu et al., 2020; Coelho et al., 2020), to the best of our knowledge, this is the only study to date that compares emotions evoked by the risk or health consequences of a pandemic with other types of threats. Finally, disgust-related stimuli in general scored as low fear-evoking (as expected), except for some modern threats depicting toxic chemical substances etc. (see below). However, it could be argued that previous research comparing old and new threats has typically not focused on specific emotions and their intensity, but rather on other parameters such as attention, stimulus detection, or conditioning (for a review, see Shapouri and Martin, 2022). Thus, it would not be so surprising that different experimental designs may yield different results.

In the case of disgust, ancestral threats, including body waste products and worms, provoked the greatest disgust responses in our experiment, as we expected according to the literature (Curtis et al., 2011). These findings align with the theory of the behavioral immune system, suggesting that disgust may have evolved as a response to stimuli posing a real threat to the survival of our pre-technological ancestors, as they are associated with potential sources of infection (Curtis and Biran, 2001; Schaller and Park, 2011). However, what was particularly intriguing was that some modern threats, which we anticipated would elicit disgust, instead triggered quite strong fear (and anger) responses. For example, toxic chemical substances and radioactivity, although invisible in the environment, evoked fear rather than disgust. This highlights the complexity of emotional responses to modern threats and suggests that the relevance of threats, as well as cognitive factors, play a significant role in shaping emotional reactions.

These findings challenge the idea that ancestral and modern threats lead to distinct emotional outcomes. Instead, they suggest that the human emotional landscape is highly adaptable and capable of responding to a wide range of threats, whether ancient or modern, with fear, disgust, or even anger.

As the extent of the study did not allow to cover all possible situations that people might be afraid of, the respondents had the opportunity to express themselves in open-ended questions at the very end of the experiment. These responses have not been statistically processed, but we present some interesting findings. The three most frequently mentioned fears were: fear of war (including specifically the war in Ukraine, Russian aggression, or the threat of nuclear war; 112 mentions), fear for the life and health of family or loved ones (110 mentions), and fear for one’s future (most often fear of not finishing school, exams, not being able to find a job, etc.; 81 mentions). While the first two categories were rather evenly represented across all ages, fear of the future was more prevalent among younger respondents. Considering the age factor and frequency of the answer “war,” these responses could be taken as a reflection of what is currently happening in the respondents’ lives, i.e., they would represent a currently relevant threat.

For disgust, there were generally fewer stimuli not covered by the main questionnaire. Immoral behavior (e.g., lying, recklessness, or selfishness; 77 mentions) was the most frequently mentioned, followed by poor hygiene (e.g., bad human smell; 62 times), and spiders (52 times) came in third; thus, rather ancestral stimuli appeared. There was no obvious age pattern, but poor hygiene and spiders were strongly prevalent among women (men were more likely to leave the question unanswered). The difference in responses regarding fear and disgust in the open-ended questions reflects the main results from the vignette assessment.

We considered the emotion of anger to be rather secondary in situations described in this research, yet high anger scores were found in some of the vignettes. By far the highest mean anger ratings were for modern threats concerning toxic chemicals and pollution and, in general, a positive correlation between disgust and anger ratings was found. A similar interactive effect of anger and disgust (that are still viewed as separate emotions) on moral judgements and decision-making was also previously reported (Salerno and Peter-Hagene, 2013; Giner-Sorolla et al., 2018).

In conclusion, our results showed stronger saliency in ancestral stimuli when rating disgust, but not for fear and anger, where currently relevant threats predominated.

### 4.2 Stimulus specificity

Our research also delved into the concept of stimulus specificity and the triggers for individual emotions, including fear, disgust, and anger. To see the pattern in emotional response to different stimuli, we employed an exploratory factor analysis (see Supplementary Table S3).

For fear, ancestral threats, such as snakes, emerged as the most specific triggers, distinct from other threats. Snake stimulus specificity was previously demonstrated many times (there is even a specific fear-evoking snake morphotype – a venomous viperid snake; Rádlová et al., 2019; Landová et al., 2020; even cross-culturally; Frynta et al., 2023). This result aligns with the concept of evolutionary preparedness, suggesting that specific stimuli associated with ancestral dangers remain potent elicitors of fear. In contrast, pandemic threats also demonstrated a high level of specificity with two rather distinct subcategories – eliciting predominantly fear or disgust (see also Troisi, 2020), indicating that the threat relevance is a critical factor in triggering different emotions. This fact might be associated with the concept of localized parasite–host co-evolutionary races claiming that humans are more vulnerable to distant pathogens coming from outsiders rather than locals, because they have had only a limited chance to develop immunity against them (Fincher and Thornhill, 2008). Thus, the global spread of unfamiliar pathogens presents a great health risk where distinctive fear and disgust responses may compensate for the non-adapted immune system.

The complexity of disgust responses became apparent when examining fear-related stimuli. Low disgust-scoring stimuli would be predicted to group together according to disgust scores. However, modern disgust stimuli, such as chemical pollution and radioactivity, did not group with other fear-related vignettes, although the disgust ratings were not very high (lower than fear scores). This suggests that the emotional responses to modern disgust threats are multifaceted, involving both fear and disgust.

Disgust results did not show such levels of specificity as no subcategories of stimuli remained separate in the analyses (as opposed to, for example, the above-mentioned snakes). It was also demonstrated in previous research, that disgust might be more prone to generalization, e.g., in harmless stimuli visually resembling primary disgust elicitors (e.g., slimy worm-like animals; Davey, 2011; Staňková et al., 2021).

Anger, an emotion often associated with frustration and irritation, demonstrated its unique patterns of specificity. Modern disgust stimuli, such as chemical pollution and radioactivity, triggered distinct anger responses, suggesting that these threats carry a moral dimension (Salerno and Peter-Hagene, 2013; Giner-Sorolla et al., 2018). Additionally, pandemic threats connected with disgust elicitors were also specific triggers of anger, highlighting the multifaceted nature of emotional reactions to global health crises (Mota et al., 2020; Pfefferbaum and North, 2020; Schwambergová et al., 2023).

### 4.3 Adaptation of psychobehavioral circuits to pandemic threats

The adaptability of psychobehavioral circuits for processing fear and disgust to pandemic threats was one of the major aims of our study. While we did not calculate the effect of pandemic threats separately, we analysed their emotional impact in comparison to other types of threats. The results demonstrated that pandemic threats elicited a range of rather high-intensity emotional responses (even after the first wave of COVID-19), including fear, disgust, and anger, yet it remains a specific category of threats. This suggests that the psychobehavioral circuits responsible for processing fear and disgust may have adapted to respond to the unique challenges posed by global health crises (McEwen and Gianaros, 2011).

Pandemic threats, particularly relevant in the context of our contemporary world, elicit a complex array of emotions (Taylor, 2019). The rapid spread and high mortality rates of infectious diseases can engender fear, as evidenced by their rankings in the fear category, they also trigger strong feelings of disgust and, notably, anger (Pfefferbaum and North, 2020), leading to behavioral changes aimed at reducing the risk of infection. Additionally, the moral dimension of pandemics, involving issues of responsibility and social behavior, can trigger anger and frustration in response to non-compliance with public health measures (Barry, 2020). This multifaceted emotional response can be attributed to several factors.

First, the immediacy and unpredictability of pandemics, as seen in events like the COVID-19 pandemic, can induce fear on a global scale. The fear of infection and the potential consequences for one’s health and well-being are palpable, leading to heightened anxiety and stress (Coelho et al., 2020; review in Salari et al., 2020). Second, the moral dimension of pandemics cannot be overlooked. The study suggests that pandemic threats, often associated with issues of public health and societal responsibility, may evoke anger (Coelho et al., 2020; Trnka and Lorencová, 2020). Factors such as government responses, misinformation, and social behavior can contribute to a sense of moral outrage. This complex interplay of emotions reflects the broader societal impact of pandemics and the ethical dilemmas they pose.

### 4.4 Implications for risk perception and decision-making

Understanding the complexity of emotional responses to threats has significant implications for risk perception and decision-making. Individuals may weigh emotional responses differently when assessing risks, and this can influence their choices and behaviors. For instance, an immediate fear response to a modern threat may lead to a heightened sense of danger, potentially affecting risk-taking behaviors. This has been previously shown in the study by Siegrist and Sütterlin (2014). The affect associated with natural or human-caused hazards influenced how people interpreted new information and mediated the evaluation o of negative outcomes associated with the hazard. In other words, equally negative outcomes are differently evaluated depending on the cause when people are more concerned with human than natural hazards. Such a cognitive-affective bias may finally lead to riskier decisions.

Recognizing the emotional dimensions of pandemic threats, including fear, disgust, and anger, can inform public health interventions and messaging. Strategies to mitigate the spread of diseases may benefit from a nuanced understanding of how people emotionally respond to pandemic-related information and directives. Our results may also indicate that humans in modern times can adequately assess current risks, even when dealing with newly emerging threats. It cannot be said that modern behavior and decision-making are entirely dependent on evolutionary processes, although in some cases the influence of evolution may still be strong - for example, in some specific phobias (e.g., snake phobia), where there is a relatively conserved intense response to ancestral danger that may be maladaptive or at least exaggerated in modern times.

### 4.5 Future directions

This study offers a thought-provoking exploration of human emotional responses to a diverse array of threats. However, it also raises numerous questions that warrant further investigation. Future research could delve deeper into the interplay of emotions in response to specific threat scenarios and explore how individual differences, cultural factors, and personal experiences shape emotional responses.

There is an opportunity for more elaboration of research on modern disgust stimuli; similar works to those on fear (except for moral disgust) are still lacking. Although in our study modern stimuli tended to elicit more of a fear response, perhaps a different design would have reached different results. Among other things, it also depends on the type of stimulation - modality: e.g., use of picture stimuli, possibly olfactory (irritating chemical smell) or even auditory (coughing), etc.

In our study, we mostly gave space to the conscious response; it would be useful to design an experiment with a greater automatic unconscious component (e.g., psychophysiology) that could, among other things, reveal more about whether the pandemic can be considered more of an ancestral or modern threat.

This paper did not show a large effect of respondents’ characteristics, but it would be useful to do further analyses if we had more data on respondents - for example, information on their sensitivity to specific threats or their emotional response in general (e.g., questionnaires measuring disgust sensitivity or trait anxiety), or to elaborate more on the effect of age for different types of threats. Finally, it would also be worth studying the effect of other than biological education on emotional evaluation of ancestral and modern threats.

## 5 Conclusion

The primary goal of this project was to gain a deeper understanding of the evolution of emotions and how evolutionarily ancestral systems of perception can function in a modern world with newly emerging threats. The threat of a pandemic forms a specific category and people process it emotionally and cognitively. Ancestral threats may not be stronger in general, there is often an effect of the current relevance of the threat, but ancestral stimuli may have a specific pattern of response. Disgust appears to be an emotion where ancestral stimuli are as strong or stronger than other tested stimuli, and the influence of disgust-inducing stimuli on the perception of pandemics cannot be rejected. We confirmed the need to consider moral aspects and anger, especially when evaluating pandemics and modern threats.

In conclusion, our study offers valuable insights into the emotional responses to ancestral and modern threats and their adaptation to pandemic challenges. The interplay between ancestral and modern threats, stimulus specificity, and the adaptability of psychobehavioral circuits highlight the complexity of human emotional responses. Our findings contribute to a deeper understanding of human psychology, shedding light on how the human brain navigates the complexities of a rapidly changing world. As we continue to encounter novel threats, our emotional responses evolve, providing valuable insights into the adaptability and resilience of the human mind. As we move forward, further exploration of emotional responses to contemporary challenges will be essential for informing fields such as psychology, evolutionary biology, and public health. This research challenges the preconceived notions about which threats should provoke the strongest emotional responses and highlights the adaptability and specificity of our emotional reactions. By embracing the complexity of human emotions, we can better navigate the ever-evolving landscape of threats and continue to adapt and thrive in the face of adversity. These insights contribute to our understanding of human psychology and behavior in an ever-changing world.

## Data availability statement

The original contributions presented in the study are included in the article/Supplementary material, further inquiries can be directed to the corresponding author.

## Ethics statement

The studies involving humans were approved by Ethics Committee of Charles University, Faculty of Science (approval no. 2021/02, granted on 14 April 2021) and National Institute of Mental Health (no. 91/21, granted 31 March 2021). The studies were conducted in accordance with the local legislation and institutional requirements. The participants provided their written informed consent to participate in this study.

## Author contributions

ŠP: Data curation, Formal analysis, Investigation, Software, Visualization, Writing – original draft, Writing – review & editing. JP: Conceptualization, Formal analysis, Methodology, Validation, Writing – original draft, Writing – review & editing. MJ: Data curation, Investigation, Writing – review & editing. AC: Investigation, Writing – review & editing. KS: Investigation, Writing – review & editing. DF: Conceptualization, Formal analysis, Methodology, Software, Supervision, Validation, Writing – review & editing. EL: Conceptualization, Funding acquisition, Methodology, Project administration, Supervision, Writing – original draft, Writing – review & editing.
