# Supplementary material for: Human emotional evaluation of ancestral and modern threats: fear, disgust, and anger
Source: Front Psychol. 2024 Jan 4;14:1321053. doi: 10.3389/fpsyg.2023.1321053 (PMC10794497; doi:10.3389/fpsyg.2023.1321053)

Supplementary Figure S1: Cluster tree diagrams based on fear (A), disgust (B) and anger (C) ratings (raw scores). The matrix was based on Pearson's correlations (1-r) and Ward's method of clustering was adopted. For the full text of vignettes, see Supplementary Table S1.

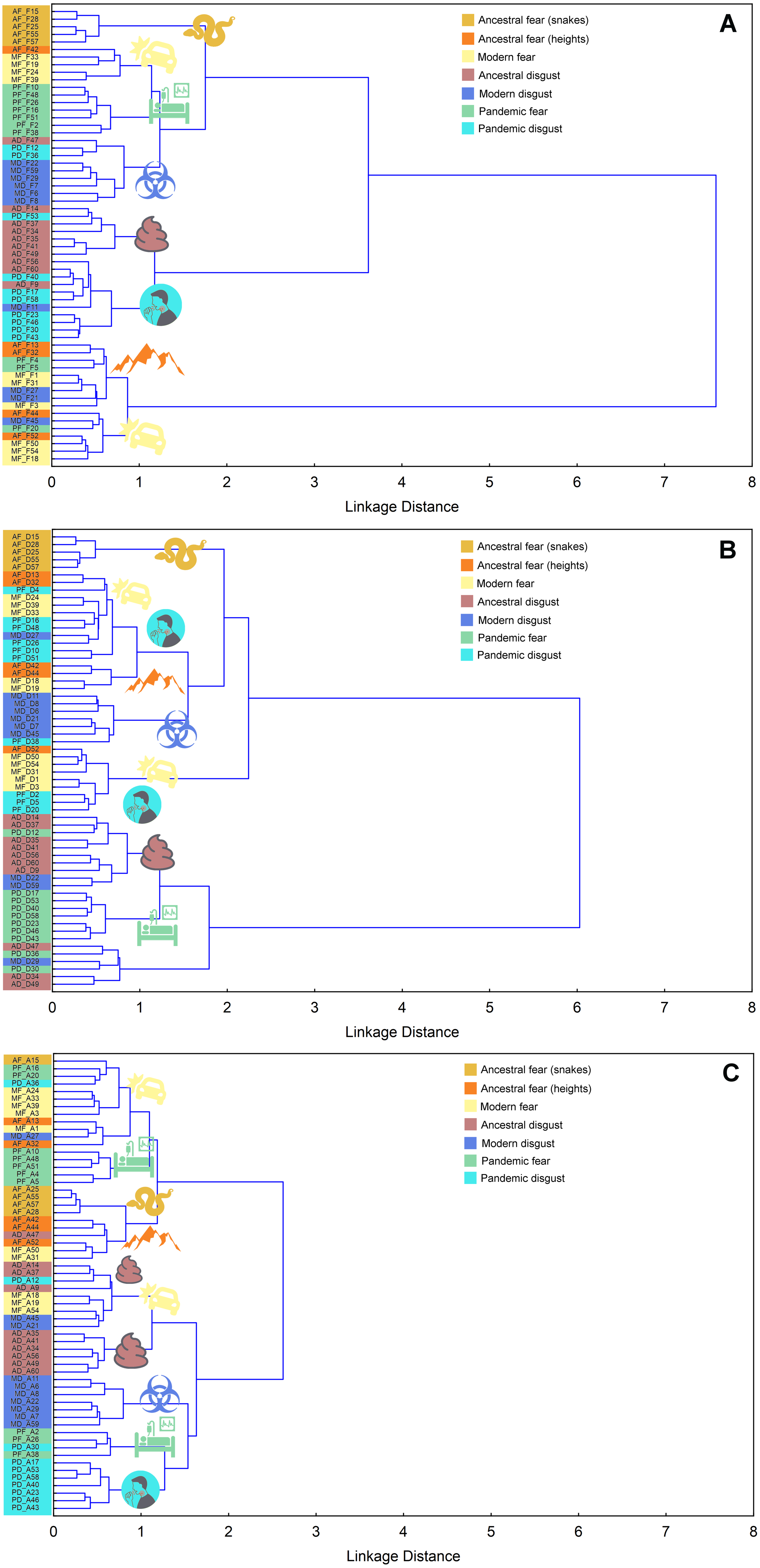

Supplement: Supplementary file 6 [file Image_1.pdf]
